# Supplementary material for: Physiologically Based Pharmacokinetic Modeling of Biologic Case Studies in Monkeys and Humans Reveals the Necessity of an Additional Clearance Term
Source: Pharmaceutics. 2025 Apr 24;17(5):560. doi: 10.3390/pharmaceutics17050560 (PMC12115253; doi:10.3390/pharmaceutics17050560)
Supplement: Supplementary file 1 [file pharmaceutics-17-00560-s001.zip › pharmaceutics-3547018-supplementary.pdf]

Supplementary Material

Article: Physiologically based pharmacokinetic modelling of biologic case studies in monkeys and humans reveals the necessity of an additional clearance term.

Authors: Felix Stader, Pradeep Sharma, Weize Huang, Mary P Choules, Marie-Emilie Willemin, Xinwen Zhang, Estelle Yau, Abdallah Derbalah, Adriana Zyla, Cong Liu, Armin Sepp

Corresponding Author: Felix Stader
Certara Predictive Technologies
Certara UK Ltd.
Sheffield, S1 2BJ, UK
felix.stader@certara.com

Table S1: Information about the seven different case studies.

No Case	Biologic drug	Route of administration	Comment on the number of simulations
Case 1	Anti-TNFα mAb	IV	-
Case 2	Half-life extended mAb	IV	-
Case 3	IgG-based mAb	IV	2 different doses
Case 4	Anti-PD-L1 mAb	IV	4 different mAbs
Case 5	Anti-IL23 mAb	IV	5 different doses
Case 6	Anti-Nectin 4 ADC	IV	2 different doses
Case 7	7 different Bi-TCE	SC	7 different Bi-TCE

ADC = antibody-drug conjugate, Bi-TCE = bispecific T-cell engager, IgG = immunoglobulin G, IL = interleukin 23, IV = intravenous, mAb = monoclonal antibody, PD-L1 = programmed cell death ligand 1, SC = subcutaneous, TNF = tumor necrosis factor.

Table S2: Information about the used literature studies in cynomolgus monkeys.

biologic drug	doses	reference
Humanized anti-VEGF mAb	2 mg/kg<br>10 mg/kg<br>50 mg/kg	[1]
Humanized anti-IL17 mAb	1 mg/kg<br>3 mg/kg<br>10 mg/kg	[2]
Humanized anti-IL13 mAb	1 mg/kg<br>3 mg/kg<br>10 mg/kg	[3]
Humanized anti-IL13 mAb	1 mg/kg<br>100 mg/kg	[4]
Belumimab	5 mg/kg<br>50 mg/kg<br>150 mg/kg	[5]
Humanized anti-TNFα mAb	5 mg/kg	[6]
Humanized anti-RSV mAb	1 mg/kg<br>50 mg/kg<br>200 mg/kg	[7]
IgG-based mAb	1 mg/kg	[8]
Humanized anti-hepatitis B virus mAb	1 mg/kg	[9]
Humanized anti-CD154 mAb	20 mg/kg	[10]
Humanized anti-RSV mAb	30 mg/kg	[11]
Humanized anti-VEGF mAb	5 mg/kg	[12]
Humanized anti-PCSK9 mAb	1 mg/kg	[13]
Humanized anti-IL23 mAb	3 mg/kg	[14]

CD154 = cluster of differentiation 154, IgG = immunoglobulin G, IL = interleukin, mAb = monoclonal antibody, PCSK9 = proprotein convertase subtilisin/kexin type 9, RSV = respiratory syncytial virus, TNF = tumor necrosis factor, VEGF = vascular epithelial growth factor.

Reference

1. Lin, Yvonne S., Cindy Nguyen, Jose-Luis Mendoza, Enrique Escandon, David Fei, Y. Gloria Meng, and Nishit B. Modi. "Preclinical Pharmacokinetics, Interspecies Scaling, and Tissue Distribution of a Humanized Monoclonal Antibody against Vascular Endothelial Growth Factor." *The Journal of pharmacology and experimental therapeutics* 288, no. 1 (1999): 371-78.
2. Han, Chao, George R. Gunn, Joseph C. Marini, Gopi Shankar, Helen Han Hsu, and Hugh M. Davis. "Pharmacokinetics and Immunogenicity Investigation of a Human Anti-Interleukin-17 Monoclonal Antibody in Non-Naïve Cynomolgus Monkeys." *Drug Metabolism and Disposition* 43, no. 5 (2015): 762-70.
3. Nnane, Ivo P., Zhenhua Xu, Honghui Zhou, and Hugh M. Davis. "Non‐Clinical Pharmacokinetics, Prediction of Human Pharmacokinetics and First‐in‐Human Dose Selection for Cnto 5825, an Anti‐Interleukin‐13 Monoclonal Antibody." *Basic & Clinical Pharmacology & Toxicology* 117, no. 4 (2015): 219-25.
4. Vugmeyster, Yulia, Pamela Szklut, Lioudmila Tchistiakova, William Abraham, Marion Kasaian, and Xin Xu. "Preclinical Pharmacokinetics, Interspecies Scaling, and Tissue Distribution of Humanized Monoclonal Anti-Il-13 Antibodies with Different Il-13 Neutralization Mechanisms." *International immunopharmacology* 8, no. 3 (2008): 477-83.
5. Halpern, Wendy G., Patrick Lappin, Thomas Zanardi, Wendy Cai, Marta Corcoran, John Zhong, and Kevin P. Baker. "Chronic Administration of Belimumab, a Blys Antagonist, Decreases Tissue and Peripheral Blood B-Lymphocyte Populations in Cynomolgus Monkeys: Pharmacokinetic, Pharmacodynamic, and Toxicologic Effects." *Toxicological sciences* 91, no. 2 (2006): 586-99.
6. Deng, Rong, Kelly M. Loyet, Samantha Lien, Suhasini Iyer, Laura E. DeForge, Frank-Peter Theil, Henry B. Lowman, Paul J. Fielder, and Saileta Prabhu. "Pharmacokinetics of Humanized Monoclonal Anti-Tumor Necrosis Factor-A Antibody and Its Neonatal Fc Receptor Variants in Mice and Cynomolgus Monkeys." *Drug Metabolism and Disposition* 38, no. 4 (2010): 600-05.
7. Davis, Charles B., Timothy W. Hepburn, James J. Urbanski, Deborah C. Kwok, Timothy K. Hart, Danuta J. Herzyk, Sandra G. Demuth, Michelle Leland, and Gerald R. Rhodes. "Preclinical Pharmacokinetic Evaluation of the Respiratory Syncytial Virus-Specific Reshaped Human Monoclonal Antibody Rshz19." *Drug Metabolism and Disposition* 23, no. 10 (1995): 1028-36.
8. Hinton, Paul R., Mary G. Johlfs, Joanna M. Xiong, Kelly Hanestad, Kelly C. Ong, Chuck Bullock, Stephen Keller, Meina Tao Tang, J. Yun Tso, and Max Vásquez. "Engineered Human Igg Antibodies with Longer Serum Half-Lives in Primates." *Journal of Biological Chemistry* 279, no. 8 (2004): 6213-16.
9. Hinton, Paul R., Joanna M. Xiong, Mary G. Johlfs, Meina Tao Tang, Stephen Keller, and Naoya Tsurushita. "An Engineered Human Igg1 Antibody with Longer Serum Half-Life." *The Journal of Immunology* 176, no. 1 (2006): 346-56.
10. Ferrant, Janine L., Christopher D. Benjamin, Anne H. Cutler, Susan L. Kalled, Yen-Ming Hsu, Ellen A. Garber, Donna M. Hess, Renee I. Shapiro, Norma S. Kenyon, and David M. Harlan. "The Contribution of Fc Effector Mechanisms in the Efficacy of Anti-Cd154 Immunotherapy Depends on the Nature of the Immune Challenge." *International immunology* 16, no. 11 (2004): 1583-94.
11. Dall'Acqua, William F., Peter A. Kiener, and Herren Wu. "Properties of Human Igg1s Engineered for Enhanced Binding to the Neonatal Fc Receptor (Fcrn)." *Journal of Biological Chemistry* 281, no. 33 (2006): 23514-24.
12. Yeung, Yik Andy, Xiumin Wu, Arthur E. Reyes, Jean-Michel Vernes, Samantha Lien, John Lowe, Mauricio Maia, William F. Forrest, Y. Gloria Meng, and Lisa A. Damico. "A Therapeutic Anti–Vegf Antibody with Increased Potency Independent of Pharmacokinetic Half-Life." *Cancer research* 70, no. 8 (2010): 3269-77.
13. Henne, Kirk R., Brandon Ason, Monique Howard, Wei Wang, Jeonghoon Sun, Jared Higbee, Jie Tang, Katherine C. Matsuda, Ren Xu, and Lei Zhou. "Anti-Pcsk9 Antibody Pharmacokinetics and Low-Density Lipoprotein-Cholesterol Pharmacodynamics in Nonhuman Primates Are Antigen Affinity–Dependent and Exhibit Limited Sensitivity to Neonatal Fc Receptor–Binding Enhancement." *The Journal of pharmacology and experimental therapeutics* 353, no. 1 (2015): 119-31.

14. Köck, K., W. J. Pan, J. M. Gow, M. J. Horner, J. P. Gibbs, A. Colbert, T. J. Goletz, K. J. Newhall, W. A. Rees, and Y. Sun. "Preclinical Development of Amg 139, a Human Antibody Specifically Targeting Il‐23." *British Journal of Pharmacology* 172, no. 1 (2015): 159-72.
